# Beyond Clinical Trials in Patients With Multiple Myeloma: A Critical Review of Real-World Results

**DOI:** 10.3389/fonc.2022.844779

**Published:** 2022-05-11

**Authors:** Luca Bertamini, Giuseppe Bertuglia, Stefania Oliva

**Affiliations:** SSD Clinical Trial in Oncoematologia e Mieloma Multiplo, Division of Hematology, University of Torino, Azienda Ospedaliero-Universitaria Città della Salute e della Scienza di Torino, Torino, Italy

**Keywords:** multiple myeloma, clinical trials, real world, immunomodulatory drugs, proteasome inhibitors, monoclonal antibodies, small molecules, immunotherapy

## Abstract

The current strategies for the treatment of multiple myeloma (MM) have improved, thanks to effective drug classes and combination therapies, for both the upfront and relapsed settings. Clinical trials for newly diagnosed transplant-ineligible patients led to the approval of immunomodulatory drugs (IMiDs) and proteasome inhibitors (PIs) in combination with anti-CD38 monoclonal antibodies (mAbs), to be administered during the induction phase before transplantation and during maintenance treatment, with lenalidomide recommended until relapse. In relapsed/refractory patients, the complex treatment scenario currently includes several options, such as triplets with anti-CD38 mAbs plus IMiDs or PIs, and novel targeted molecules. Comparisons among clinical trials and real-world data showed a good degree of reproducibility of some important results, particularly in terms of overall response rate, progression-free survival, and overall survival. This may help clinicians towards a proper selection of the best treatment options, particularly in real-world settings. However, as compared with the management of real-world settings, clinical trials have some pitfalls in terms of outcome and especially in terms of safety and quality of life. In fact, trials include younger and presumably healthier patients, excluding those with worst clinical conditions due to MM features (e.g., renal insufficiency or bone disease, which can impair the performance status) and comorbidities (e.g., cardiac and pulmonary disease), thus resulting in a possible lack of representativeness of data about the patients enrolled. In this review, we analyze comparable and discrepant results from clinical trials vs. real-world settings published in the last 10 years, focusing on different drugs and combinations for the treatment of MM and providing an overview of treatment choices.

## 1 Introduction

In the last 25 years, the treatment possibilities for patients with multiple myeloma (MM) largely improved, thanks to the introduction of non-chemotherapeutic approaches (proteasome inhibitors [PIs] and immunomodulatory drugs [IMiDs]), monoclonal antibodies (mAbs), and, more recently, small molecules and immunotherapeutic approaches. Results from different phase II and III clinical trials including these newer agents led to a substantial revision of the therapeutical approaches for both newly diagnosed (ND)MM and relapsed/refractory (RR)MM patients in clinical practice.

Still, there are some important differences, in terms of efficacy and safety, in the management of MM patients in real-world (RW) settings and in clinical trials. First, clinical trials rely on eligibility and exclusion criteria that are mostly related to comorbidities and performance status. This leads to patient selection and clinical characteristics that differ from those in clinical practice. For instance, severe renal insufficiency is an important prognostic factor observed in around 10% of patients at diagnosis (1, 2) and is also an exclusion criterion in many clinical trials. Second, the treatment scenario for RRMM patients in the RW can differ from that in clinical trials, in which some therapies are used in the earliest MM lines (1-3 lines), thus resulting in substantial discrepancies in terms of response and outcome.

Obviously, RW studies have many shortcomings, since they are mostly small, single-center, retrospective data collections. Moreover, RW data usually report shorter ranges of median values for progression-free survival (PFS) and time-to-next treatment (TTNT) than those observed in phase III trials.

Nonetheless, there is increasing interest in collecting RW data. In the EMMOS observational study (2), retrospective data from 2358 patients from 22 countries were collected, including patients treated with or without autologous stem-cell transplantation (ASCT) at diagnosis. Nowadays, other large RW studies are ongoing: the LocoMMotion is exploring the outcome of patients receiving at least an IMiD, a PI, and an anti-CD38 mAb as treatment (3); the KarMMa-RW retrospectively enrolled a matched cohort to be compared with patients receiving chimeric antigen receptor (CAR) T-cell immunotherapy with idecabtagene vicleucel; and the prospective RW study INSIGHT-MM will enroll around 3000 patients (4).

In this review, we compare data from the RW setting with results from clinical trials, focusing on drugs used both in the NDMM and RRMM settings. Moreover, we will briefly report early RW data about novel drugs under investigation or waiting for approval.

## 2 Immunomodulatory Drugs

### 2.1 Lenalidomide

Lenalidomide is an IMiD with pleiotropic effects and without a fully known mechanism of action (5). Today, lenalidomide is approved in combination with dexamethasone (Rd) as a standard-of-care option for the treatment of transplant-ineligible (NTE) patients. Moreover, lenalidomide has been approved in combination with bortezomib-dexamethasone (VRd) as induction treatment before ASCT (6), as maintenance therapy after ASCT (7), and in combination with other drugs in RRMM (8–11). Recently, the addition of daratumumab to Rd (Dara-Rd) in NTE patients showed superiority over Rd and has become a new standard of care (12).

#### 2.1.1 Lenalidomide-Based Combinations as First-Line Therapy in Transplant-Ineligible Patients: Real-World Comparisons With Proteasome Inhibitors

At diagnosis, the treatment options for patients not eligible for ASCT due to age or comorbidities have evolved over the last years. The standards of care for this patient population are a fixed-duration treatment strategy with 9 cycles of bortezomib-melphalan-prednisone (VMP), Rd until progression or intolerance, or 6 cycles of bortezomib-Rd (VRd) followed by Rd until progression or intolerance. These regimens have never been directly compared, and their approval comes from three randomized phase III trials [FIRST (13), VISTA (14), and SWOG S0777 (15)] with similar efficacy, PFS, and overall survival (OS) rates (see **Table 1**). Regarding carfilzomib (K), the comparison of VRd and KRd was tested in the ENDURANCE trial in NDMM patients without an immediate intent for ASCT, and KRd did not show superiority in terms of PFS (17).

**Table 1:** Major clinical trials and real-world studies based on lenalidomide in first-line treatment.

Setting	Study	Age*	Prior therapy	Response ORR/≥VGPR/≥CR	>PFS/OS/DOT/TTP	Toxicity, G≥3
**MAJOR CLINICAL TRIALS**						
**First line,** **NTE patients**	**FIRST** (13, 16) Rd vs MPT: N=1623 Rd (until PD): 535 Rd (18 cycles): 541	73	–	Rd (until PD): - 75%/44%/15% Rd (18 cycles): - 73%/43/14%	Rd (until PD): - mPFS: 26 mo - mOS: 59 mo Rd (18 cycles): - mPFS: 21 mo - mOS: 62 mo	Rd (until PD): - Anemia 18% - Neutropenia 28% - Infection 29% - Pneumonia 8% Rd (18 cycles): - Anemia 16% - Neutropenia 26% - Infection 22% - Pneumonia 8%
**First line,** **NTE patients**	**MAIA** (12) Dara-Rd vs. Rd, N=737	73	–	Dara-Rd: - 92%/79%/48% Rd: - 81%/53%/25%	Dara-Rd: - PFS: 32 mo - mOS: NR (FU 28 mo) Rd: - mPFS: 32 mo - mOS: NR (FU 28 mo)	Dara-Rd vs. Rd: - Anemia 11% vs. 19% - Neutropenia 50% vs. 35% - Infection 32% vs. 23% - Pneumonia 14% vs. 8%
**First line,** **NTE patients**	**SWOG S0777** (15) VRd vs. Rd, N=525	63	–	VRd vs. Rd: - 82%/43%/16% - 72%/32%/86%	VRd vs. Rd: - mPFS: 43 vs. 30 mo - mOS: 75 vs. 64 mo	VRd vs. Rd: - Hematologic AEs 47% vs. 32% - Infection 14% vs. 14%
**First line,** **NTE patients**	**ENDURANCE** (17) VRd vs. KRd, N=1053	64	–	VRd: - 84%/65%/15% KRd: - 87%/74%/18%	VRd vs. KRd: mPFS: 34.4 vs. 36.6 mo mOS: NR vs. NR 3-y OS rate: 86% vs. 84%	- PN 8% vs. <1% - VTE 2% vs. 5%
**First line,** **TE patients**	**IFM 2009** (18) VRd-ASCT-VRd: N=350	60	–	- 98%/88%/59%	mPFS: 50 mo mOS: NR 4-y OS rate: 81%	- Neutropenia 92% - Anemia 20% - Thrombocytopenia 83% - Infection 20% - PN 13% - SPM 1.5%
**Post-ASCT maintenance**	**RV-MM-PI-209** (19) R vs. no R: N=251	57	Len-exposed: 100%	–	mPFS: 41.9 mo 3-y OS rate: 88%	- Neutropenia 27% - Infection 7% - Dermatologic AEs 5% - Discontinuation 6% R vs. no R: - SPM 5% vs. 5%
**Post-ASCT maintenance**	**IFM2005-02** (20) R vs. placebo: N=614	55	Bort-exposed: 92%	–	mPFS: 41 mo 4-y OS rate: 73%	- Discontinuation 27.1% R vs. no R: - SPM 8% - 5-y SPM 3.1% vs 1.2%
**Post-ASCT maintenance**	**CALGB 100104** (7) R vs. placebo: N =460	59	Bort-exposed: 42% Len-exposed: 34%	–	TTP: 46 mo 3-y OS rate: 88%	- Discontinuation 9% R vs. no R: - SPM 8% vs. 3%
**Post-ASCT maintenance**	**McCarthy et al., 2017: meta-analysis** (21) R vs. placebo/observation: N=1208	58	Bort-exposed: 39% Len-exposed: 22%	–	PFS: 52.8 mo 7-y OS rate: 62% mOS: NR	- Discontinuation 29.1% R vs. no R: - Hematologic SPM 5.3% vs. 0.8%** - Solid SPM 5.8% vs. 2%**
**Post-ASCT maintenance**	**Myeloma XI** (22) R vs. observation: N=1981	66	Len-exposed: 63%	–	mPFS: 39 mo 5-y OS: 61 mo	- Neutropenia 33% - Discontinuation 52% (28% due to AE) R vs. no R: - SPM 2.4% vs. 1.4%
**MAJOR REAL-WORLD STUDIES**						
**First line,** **NTE patients**	**Cejalvo et al., 2021** (23) Rd: N=24 Other: N=651	75.6	–	–	mPFS: 20 mo mOS: 34.6 mo	NA
**First line,** **NTE patients**	**Canadian Myeloma Research Group database (CMRG-DB), Jimenez-Zepeda et al., 2021** (24) Rd: N=208 Other: N=948	75	–	- 87%/61%/28%	mPFS: 28 mo mOS: 66 mo	NA
**First line,** **NTE patients**	**Zamagni et al., 2021** (25) Rd: N=194 Other: N=233	74	–	–	mPFS: 38 mo mOS: NA	NA
**First line,** **NTE patients**	**Chari et al., 2019** (26) Rd: N=814 VRd: N=319	Rd vs. VRd: 70 vs. 64	–	–	Rd vs. VRd: - mTTNT: 36.7 vs. 37.5 mo - DOT: 12 vs. 14.8 mo	NA
**First line,** **NTE patients**	**EMMY, Decaux et al., 2021** (27) Rd: N=162 VRd: N=158	Rd vs. VRd: 79.6 vs. 69.3	–	–	Rd vs. VRd: - mTTNT: 29 vs. 24 mo - mTTNT, age <75 y: 29.4 vs. 34.7 mo - mTTNT, age ≥75 y: 29.5 vs. 17.1 mo - 2-y OS rate: 86% vs. 85%	NA
**First line,** **TE patients**	**Joseph et al., 2020** (28) N=1000	61	–	- 97%/90%/33%	mPFS: - 65 mo - ISS I vs. III: 73 vs. 50 mo - R-ISS I vs. III: 129 vs. 31 mo mOS: - 127 mo - ISS I vs. III: 89 vs. 95 mo - R-ISS I vs. III: NR vs. 60 mo	NA
**Post-ASCT maintenance**	**Canadian Myeloma Research Group database (CMRG-DB), Cherniawsky et al., 2021** (29) R vs. no R: N=723 vs. 533	NA	Bort-exposed: 100% Len-exposed: 1%	–	mPFS: 58.2 mo 5-y OS rate: 81% mOS: NR (>124 mo)	- Discontinuation 20% R vs. no R: - SPM 3.4% vs. 6.4%
**Post-ASCT maintenance**	**Chakraborty et al., 2018** (30) R vs. no R: N=132 vs. 341	61	PI-based: 34% IMiD-based: 26% PI+IMiD-based: 40%	–	mPFS: 36.5 mo 4-y OS rate: 79% - R: ISS I/II vs. III, OS rate: 85% vs. 73% - No R: ISS I/II vs. vs. III, OS rate: 84% vs. 68%	- Discontinuation 17% R vs. no R: 7-y SPM 5.4% vs. 6.4%
**Post-ASCT maintenance**	**Connect MM Registry, Jagannath et al., 2018** (31) R vs. no R: N=213 vs. 165*** Other maintenance therapies: 54	60	Bort-exposed: 97% Len-exposed: 45%	–	mPFS: 50.3 mo 3-y PFS rate: 50.8% mOS: NR 3-y OS rate: 85%	- Discontinuation 19% R vs. no R: - SPM 4.7% vs. 6.1%

*Age: median age. **Frequency evaluated before progressive disease (follow-up: 70.5 months). ***Induction strategies and percentage calculated for the entire cohort population (N=432).

RW, real world; TE, transplant-eligible; NTE, non-transplant-eligible; ASCT, autologous stem-cell transplantation; R, Len, lenalidomide; d, dexamethasone; Dara, daratumumab; V, Bort, bortezomib; K, carfilzomib; ORR, overall response rate; ≥VGPR, at least a very good partial response; ≥CR, at least a complete response; PD, progressive disease; NA, not available; mo, months; y, years; FU, follow-up; NR, not reached: PI, proteasome inhibitor; IMiD, immunomodulatory drug; PFS, progression-free survival; OS, overall survival; mPFS, median PFS; mOS, median OS; DOT, duration of treatment; TTNT, time to next treatment; mTTNT, median TTNT; ISS, International Staging System stage; R-ISS, Revised ISS stage; G, grade, PN, peripheral neuropathy; VTE, venous thromboembolism; SPM, second primary malignancy.

One of the unsolved questions in this patient setting is whether Rd and VMP represent the best backbone. Up to now, no clinical trials compared these regimens, and cross-trial comparisons did not show important differences. Renal toxicity associated with lenalidomide limits its use in patients with important renal failure at diagnosis, although it may be well tolerated due to its oral administration. The advantages of VMP are the rapid efficacy, the safe use in case of renal insufficiency, and the higher efficacy in patients with adverse cytogenetic risk, while the main drawback is represented by the neurotoxicity associated with bortezomib. Nowadays, a randomized clinical trial (REAL-MM) is comparing Rd and VMP and is probably going to solve some of these issues, shedding light on which regimen better fits each patient subpopulation.

RW heterogeneity reflects this absence of detailed guidelines regarding patient subpopulations that can help clinicians select the most appropriate treatment. In the EMMOS study, first-line treatment consisted of bortezomib-based therapy without IMiDs in around half of patients, while a small proportion of patients received thalidomide/lenalidomide-based therapy without bortezomib (18%), and only 4% an IMiD plus bortezomib (2). Similarly, in another retrospective, multicenter, observational study of NTE patients conducted in Spain, the lenalidomide-based combinations, particularly Rd, were less used than the bortezomib-based combinations. Interestingly, patients treated with VMP and Rd showed lower survival rates than those in patients enrolled in clinical trials, since NTE patients in the RW tend to be older and present with higher risk and worse health status than those enrolled in randomized controlled trials (RCTs) (23). Moreover, in an analysis based on the Canadian Myeloma Research Group database (CMRG-DB), there was a clear preference for treating high-risk individuals with bortezomib-based triplets and, in particular, with bortezomib-cyclophosphamide-dexamethasone (VCd), which was the most widely used frontline bortezomib-containing regimen. Nevertheless, the median PFS was longer in patients treated with Rd than in those treated with bortezomib. In terms of survival, a (non statistically significant) trend towards a longer OS was observed with Rd, as compared with VCd (not with VMP) (24).

Zamagni et al. compared the use of lenalidomide-based and bortezomib-based regimens in eight European countries, showing that both types of treatment were equally administered (lenalidomide 48.6% and bortezomib 51.4%) and that Rd was also used in patients with renal impairment by reducing the lenalidomide dose. As compared with bortezomib-based regimens, Rd treatment was associated with longer PFS, longer time to second-line treatment and third-line treatment, but also with higher treatment-associated costs (24, 25), which should be taken into consideration by clinicians.

VRd is another approved and appealing regimen for transplant-eligible (TE) patients (15). Although VRd was superior to Rd in the clinical trial setting, there are controversies regarding its possible toxicity in the elderly population in terms of polyneuropathy and increased infections. Indeed, the SWOG S0777 trial was not focused on an elderly population and it did not consider the frailty status. A possible approach to the treatment of these patients can be the “VRd-lite” therapy, with reduced dose (lenalidomide from 25 to 15 mg) and intensity (weekly bortezomib), which showed good toxicity profile and response in a phase II trial (32). A RW study showed that elderly/NTE patients receiving VRd were younger than patients receiving Rd and that a longer duration of treatment (DOT) was associated with a longer TTNT. Health care costs related to Rd treatment were comparable with VRd and VCd treatments (26). In the EMMY study, the Rd and VRd treatment strategies improved the TTNT in NTE patients, as compared with other regimens (27). In detail, VRd should be preferred in NTE patients aged <75 years, while Rd remains highly effective in patients aged ≥75 years (**Table 1**).

Recently, two randomized phase III trials compared a backbone treatment containing VMP or Rd with these same regimens plus the anti-CD38 mAb daratumumab (Dara) (12, 33). The superiority of the daratumumab-based regimens was impressive, leading to the definition of new standards of care with Dara-VMP (ALCYONE trial) and Dara-Rd (MAIA trial). A subanalysis of the MAIA trial showed that the triplet Dara-Rd was well tolerated in frail patients, as compared with Rd (34). However, there are no data from the RW on the use of Dara-Rd as first-line treatment. Of note, a report compared, with an anchored indirect treatment comparison, data on the use of Dara-Rd in the MAIA trial to RW health registry data on elderly NTE patients treated with Rd or VRd. Within the limitations of this comparison, VRd was not associated with improved survival, as compared with Rd (hazard ratio [HR] 0.80, 95% confidence interval [CI] 0.62-1.02, *P*=0.08), and was inferior to Dara-Rd treatment in the MAIA trial (HR 0.68, 95% CI 0.48-0.98, *P*=0.04) (35). Aside from these findings, more data on the use of daratumumab in the RW setting are needed to confirm the substantial data in the MAIA trial. In particular, data on safety and on frail patients may corroborate the role of the Dara-Rd triplet as the best first-line therapy for elderly patients.

In the future, the use of VRd will probably be reconsidered in light of the results of ongoing trials investigating quadruplets containing anti-CD38 mAbs plus VRd in NTE patients: the phase III trials CEPHEUS (Dara-VRd vs. VRd) and IMROZ (isatuximab-VRd vs. VRd).

#### 2.1.2 Lenalidomide as First-Line Therapy in Transplant-Eligible Patients

Among the different drugs approved for induction treatment before high-dose chemotherapy plus ASCT, lenalidomide is used in combination with dexamethasone and bortezomib (VRd). The IFM 2009 trial showed the superiority of VRd as induction treatment followed by ASCT vs. only VRd in terms of PFS and overall response rate (ORR), while no difference was observed in terms of 4-year OS. Perrot et al. presented an updated analysis of this trial, reporting the long-term outcome in the two arms and the impact of second-line treatments on PFS2 and OS: with a follow-up of almost 8 years, the median OS was not reached (NR) and there was no difference between the two treatment strategies in terms of PFS2 and OS. Minimal residual disease (MRD) was the most important predictor of outcome because, as already noted in the analysis of the IFM 2009, PFS was longer in patients who achieved MRD negativity than in those who did not. In this view, MRD might be used after induction to identify those patients who probably do not require transplant (18).

Joseph at al. published the results of a large RW study enrolling 1000 consecutive patients treated with VRd as first-line therapy (28). In this cohort of RW patients, 30% of them were >65 years old, a group that is often excluded from clinical trials for TE patients. Moreover, a high number of African American (AA) patients, who are usually underrepresented in clinical trials, was included. In this cohort, despite these discrepancies between the clinical trial and RW settings, the results were comparable in terms of PFS, ORR, and OS. Important conclusions that emerged from the study analyzed by Joseph et al. were that age was not an independent predictor of shorter PFS and that no differences between non-AA and AA patients were observed, while International Staging System (ISS) and Revised ISS (R-ISS) stages retained their prognostic values, with shorter PFS and OS observed in ISS-III and R-ISS III patients than in ISS-I and R-ISS I patients. Tan et al. investigated the safety profile of VRd in another RW cohort, highlighting an important rate of worsening neuropathy or new-onset pre-existing neuropathy (56%, with 5% of grade [G]≥3). Of note, these findings did not differ from those observed in the IFM 2009 trial (36).

#### 2.1.3 Lenalidomide as Maintenance Treatment After ASCT

In several studies, treatment with lenalidomide after ASCT showed benefits in terms of PFS and OS over placebo. A recent meta-analysis based on three RCTs (7, 19, 20) included more than 1200 patients and showed the benefits of lenalidomide maintenance over placebo or observation (PFS: 52.8 vs. 23.5 months; 7-year OS: 62% vs. 50%) (21). In this meta-analysis, although cytogenetic data were not available for the majority of patients, a benefit in terms of PFS (not of OS) was observed in patients with high-risk cytogenetics in the lenalidomide group. An advantage in terms of PFS with lenalidomide maintenance vs. observation in patients with high-risk cytogenetics was observed in the phase III Myeloma XI clinical trial [HR: 0.45, 95% CI 0.33-0.62) (22).

Based on the analysis of RW data, the use of lenalidomide in maintenance treatment should be encouraged in clinical practice, especially in high-risk patients.

In a Canadian retrospective, observational study conducted by Cherniawsky at al., maintenance treatment with lenalidomide alone (LM) was superior to no maintenance treatment (NLM) in terms of PFS and OS. The benefit of LM vs. NLM was also observed in patients with high-risk cytogenetics (PFS: 53 vs. 22 months; OS, NR vs. 45.3 months, respectively) (29).

Similar results were confirmed by another RW study performed at the Mayo Clinic, in which LM was associated with a superior PFS, as compared with NLM (median PFS: 36.5 vs. 27.7 months, respectively), also in subgroups of patients with ISS stage III disease (median PFS: 40 vs. 24 months) and high-risk cytogenetics (median PFS: 27 vs. 16 months). Nevertheless, the 4-year OS rates were 79% vs. 80% (*P*=0.704). In a subgroup analysis, the 4-year OS rate in the NLM group was significantly inferior in patients with ISS stage III than in patients with stage I/II disease, but no significant difference was observed in the 4-year OS rates in the LM group. This could suggest that LM particularly benefited patients with ISS stage III disease and mitigated the adverse prognostic features of high-risk genetics and advanced ISS stage (30). These data were confirmed in another RW study performed using Connect MM, the largest non-interventional US-based prospective registry of patients with NDMM. In this study, patients with high-risk disease who received LM had significantly longer PFS vs. those who did not (median PFS: 54.5 vs. 25.7 months), but this difference did not reach significance in patients with standard-risk disease (median PFS: 50.3 vs. 33.4 months) (31).

Besides the improvement in terms of PFS, OS, and tolerable toxicity with the use of LM, findings from all clinical trials containing lenalidomide assessed the association between the use of lenalidomide and the major risk of developing second primary malignancies (SPMs), probably due to its immunomodulatory effects (7, 20, 21). Surprisingly, this major risk in the LM group was not reported in any of the three RW studies mentioned above (29–31) (**Table 1**). However, due to the retrospective nature of these studies, the long-term follow-ups of patients had the potential to under-represent SPMs.

Finally, RCT results remarkably showed that, although the risk of developing SPMs was higher in the LM group than in the NLM group, the risk of progressive disease (PD) without LM was even greater (21).

### 2.2 Pomalidomide

The third generation IMiD pomalidomide is similar to thalidomide and lenalidomide, with anti-apoptotic, anti-angiogenic, and immunomodulatory activities. At present, following the results of two phase III trials (MM-003 and STRATUS (MM-010)) (37, 38), pomalidomide is authorized in combination with dexamethasone (Pd) for the treatment of RRMM patients resistant or refractory to lenalidomide. In a population of lenalidomide- and bortezomib-exposed patients – most of whom were refractory to both drugs (75%-80%) – treatment with Pd was associated with a low ORR of ~32%, which translated into a median PFS of 4-4.6 months and a median OS of 11-11.9 months. In a subgroup analysis, response and survival rates were similar, regardless of prior refractoriness to lenalidomide and/or bortezomib, number of prior lines of therapy, cytogenetic risk, and presence of moderate renal impairment at study enrollment. RW retrospective studies confirmed the efficacy and survival outcomes of Pd reported in these two large clinical trials (39–44). Indeed, response rates were similar or slightly higher in the RW studies than in the trials, ranging from 32% to 52%. Likewise, PFS and OS were similar to those observed in the trials, with a trend toward better outcomes (median PFS of 3.4-10 months and median OS of 13-16 months; **Table 2**).

**Table 2:** Major clinical trials and real-world studies based on pomalidomide for the treatment of RRMM.

Study	Age*	Median lines (range)/prior exposure	ResponseORR/≥VGPR/≥CR	PFS/OS/TTP	Toxicity, G≥3
**MAJOR CLINICAL TRIALS**					
**MM-003** (38) Pd: N=302**	64	5 (2–14)/ Len-exposed: 100% Bort-exposed: 100% Len-ref: 95% Bort-ref: 79% PI+IMiD-ref: 75%	- 31%/6%/1%	mPFS: 4 mo mOS: 12 mo	- Neutropenia 48% - Anemia 33% - Thrombocytopenia 22% - Pneumonia 13% - Fatigue 5% - Dose reduction 27% (4% due to AE) - Dose interruption 67%
**MM-002** (45) Pd: N=103**	63	5 (1-13)/ Len-exposed: 100% Bort-exposed: 100% PI+IMiD-ref: 66%	- 33%/3%/3%	mPFS: 4.2 mo mOS: 14.4 mo	- Neutropenia 33% - Anemia 22% - Thrombocytopenia 19% - Pneumonia 22% - PN 0%
**STRATUS (MM-010)** (37) Pd: N=682	66	5 (2-18)/ Bort-exposed: 100% Len-exposed: 100% Len-ref: 95% Bort-ref: 83% PI+IMiD-ref: 80%	- 33%/8%/1%	mPFS: 4.6 mo mOS: 11.9 mo	- Neutropenia 49% - Anemia 33% - Thrombocytopenia 24% - Pneumonia 13% - Fatigue 6% - PN 2% (any G 20%) - Dose reduction 22% - Dose interruption 66% - Dose discontinuation 6% (due to AE)
**MAJOR REAL-WORLD STUDIES**					
**‘Rete Ematologica** **Pugliese E Basilicata’, Mele et al., 2019** (39) Pd: N=103	73	3 (NA)/ Last treatment received: - Bort-based regimens 29% - Len-based regimens 50%	- 51%/11%/2%	mTTP: 10 mo mOS: 16 mo	- Neutropenia 32% - Anemia 10% - Thrombocytopenia 9% - Fatigue 6% - Infection (including pneumonia) 4% - Dose reduction 27% (due to AE) - Dose discontinuation 55% (due to AE)
**Scott et al., 2018** (40) Poma-based regimens: N=87 Pd: 64%	65	5 (1-15)/ Len-ref: 90% Bort-ref: 77% PI+IMiD-ref: 69%	- 32%/7%/2%	PFS: 3.4 mo mOS: 7.5 mo	Severe AEs: - Febrile neutropenia 21% - Pneumonia 21% - Anemia 14% - Dose reduction 10%
**Polish Myeloma Group, Charlinski et al., 2018** (41) Pd: N=41 PVd: N=9	63	4 (2-8)/Bort-exposed 100% Len-exposed 98%	- 39.1%/8.7%/2.2%	PFS: 10 mo mOS: 14 mo	- Neutropenia 24% - Anemia 8% - Thrombocytopenia 10% - Respiratory tract infection 14% - PN 4% (any G 8%) - Dose reduction 18%
**The Royal Marsden Hospital, Sriskandarajah et al., 2017** (42) Pd: N=30 CPd: N=9	59	4 (1-8)/ Bort-exposed: 95% Len-exposed: 100%	- 41%/10%/0%	PFS: 5.2 mo mOS: 13.1 mo	- Neutropenia 61% - Anemia 2.5% - Thrombocytopenia 33% - Respiratory tract infection 28% - Febrile neutropenia 10% - Dose interruption/reduction 44%
**Maciocia et al., 2017** (43) Pd: N=70 Pd triplet: N=15	65	3 (2–7)/ Bort-exposed: 99% Len-exposed: 100%	- 52.9%/5.7%/0%	PFS: 5.2 mo mOS: 13.7 mo	- Neutropenia 38% - Thrombocytopenia 24% - Anemia 14% - Pneumonia 17% - AKI 7% - Neutropenic sepsis 8% - Dose reduction 19%
**Gueneau et al., 2018** (44) Pd: N=63	66	2-3 in 59% of pts/ Len-exposed: 100% Bort-exposed: 100% Len-ref: 37% Bort-ref: 24% PI+IMiD-ref: 19%	- 51%/13%/3%	mPFS: NA mOS: 3.6 y from diagnosis	- Neutropenia 14% - Thrombocytopenia 2% - Anemia 0% - Infection 25% - PN 6% (any G 29%)

*Age: median age **Data reported from the Pd arm with low-dose dexamethasone (dexamethasone at 40 mg, days 1, 8, 15, and 22).

RRMM, relapsed/refractory multiple myeloma; P, pomalidomide; d, dexamethasone; N, number; C, cyclophosphamide; V, Bort, bortezomib; ref, refractory; pts, patients; Len, lenalidomide; PI, proteasome inhibitor; IMiD, immunomodulatory drug; ORR, overall response rate; ≥VGPR, at least a very good partial response; ≥CR, at least a complete response; PFS, progression-free survival; OS, overall survival; mPFS, median PFS; mOS, median OS; TTP, time to progression; mTTP, median TTP; mo, months; NA, not available; y, years; G, grade; AE, adverse event; PN, peripheral neuropathy; AKI, acute kidney injury.

In a RW Australian experience study conducted by Scott et al., OS was shorter than that reported in the clinical trials, likely due to the advanced or refractory disease and the higher prevalence of severe comorbidities. Moreover, a large proportion of patients included in this RW study would not have met the inclusion criteria of the MM-003 trial, due to cytopenia, renal impairment, advanced comorbidities, and/or poor performance status (40). Surprisingly, in this Australian study and in a multicenter Italian study conducted by Mele et al. (39), younger age was associated with worse outcome, while in large clinical trials no difference was observed between patients aged >65 vs. <65 years (38). A possible explanation for these findings is that patients aged <65 years were significantly more anemic and thrombocytopenic at the start of pomalidomide treatment, thus suggesting a more active disease or a more intensive and toxic prior treatment than in older patients. We can speculate that this is a selection bias. Indeed, in the RW setting, a salvage treatment is offered to the majority of younger patients, regardless of their comorbidities or performance status. On the contrary, patients aged >65 years with similarly advanced disease or significant cytopenia may have received palliative care rather than treatment with Pd.

The safety of the Pd combination is another important issue. The most important G≥3 adverse events (AEs) observed in the phase III MM-003 and STRATUS (MM-010) trials are reported in **Table 2**. The incidence of venous thromboembolism was low due to the thromboprophylaxis (around 1%). Infections were one of the most common causes of death (10%) and hospitalization (60%), and, overall, dose reductions and interruptions due to AEs were common (22%-27% and 67%, respectively) (37, 38). Overall, data from the RW studies reported a toxicity profile of the Pd doublet similar to that reported in the clinical trials. The frequency of hematologic AEs varied from study to study (39–44), while infections seemed to be more common in the RW, particularly pneumonia (40, 43) (**Table 2**). Of note, peripheral neuropathy was higher in a French retrospective study than in the clinical trials (37, 44), likely due to the exclusion of patients with previous neuropathy of G>2 from the clinical trial, but not from the RW treatment with Pd.

In patients with high-risk cytogenetic disease, who usually have a dismal outcome, the efficacy of Pd seemed to be similar to that in standard-risk patients. In a RW study conducted by the Polish Myeloma Group, high-risk cytogenetics, although present in a small proportion of patients (n=7), did not affect PFS (11.0 months in high-risk patients vs. 15.0 months in standard-risk patients, *P*=0.23) or OS (13.0 vs. 15.0 months; *P*=0.10) (41). Similarly, in a UK RW study conducted by Maciocia e al., high-risk cytogenetics were detected in 44% of patients (17 out of 39), but did not impact survival (median PFS: 5.1 vs. 5.2 months in standard-risk patients; median OS: 10.9 vs. 8.4 months in standard-risk patients) (43).

Considering that it is primarily excreted by the liver, pomalidomide can be effective in RRMM patients with moderate or severe renal impairment, as shown in the MM-013 trial (46). Besides, a pooled analysis (47) of patients from three trials (MM-003, MM-010, and MM-002) reported comparable response and PFS rates, but shorter OS rates in patients with creatinine clearance of 30-60ml/min than in patients without renal impairment (47). Maciocia et al. observed that, in a population with renal impairment (creatinine clearance <45 ml/min) (43), similar response and PFS rates were reported in patients with creatinine clearance <45 ml/min without increased toxicity and in patients with normal renal function, although a non-statistically significant median OS was shorter in the first group (7.4 vs. 10.1 months).

The future of pomalidomide is in combination with other novel agents. Pomalidomide-based triplets have been recently approved: pomalidomide with bortezomib-dexamethasone (PVd), elotuzumab-dexamethasone (Elo-Pd), daratumumab-dexamethasone (Dara-Pd), and isatuximab-dexamethasone (Isa-Pd) (48–51). Regarding these recently approved combinations, few data are available from RW analyses. A large RW study by Davies et al. reported data on the use of several pomalidomide-based triplets in the US, even though some of them have not yet been authorized in Europe (e.g., carfilzomib-pomalidomide-dexamethasone [KPd] and ixazomib-pomalidomide-dexamethasone [IPd]). Overall, 348 patients received a pomalidomide-based triplet, in most cases as a third or subsequent line of treatment, with favorable TTNT with PVd vs. IPd and KPd (52). In the phase III OPTIMISMM trial, PVd showed similar outcomes, with a median PFS of 11 months in a population of patients with a median of 2 prior lines of therapy (48).

In the future, together with the increasing use of these triplets in clinical practice, data from the RW setting will potentially confirm the efficacy and safety observed in clinical trials.

## 3 Proteasome Inhibitors

### 3.1 Carfilzomib

The second-generation PI carfilzomib is currently approved for the treatment of RRMM patients after at least 1 line of therapy, in combination with dexamethasone (Kd) or lenalidomide-dexamethasone (KRd). A clear superiority in terms of PFS and OS was reported in two phase III randomized clinical trials: the ENDEAVOR trial compared Kd vs. bortezomib-dexamethasone (Vd; PFS: HR 0.53, 95% CI 0.44–0.65; *P*<0.0001; OS: HR 0.76, 95% CI 0.63-0.92; *P*=0.0017) (53, 54) and the ASPIRE trial compared KRd vs. Rd (PFS: HR 0.66, 95% CI 0.55-0.78, *P*=0.001; OS: HR 0.79, 95% CI 0.67-0.95; *P*=0.0045) (55).

An important limit to the use of carfilzomib is associated with its safety profile. Data from the ASPIRE and ENDEAVOR trials showed a higher occurrence of cardiovascular (CV)AEs in the carfilzomib arms, mainly hypertension (17%-32%), heart failure (7%-12%), and ischemic heart disease (4%-7%). This toxicity issue could limit its use in patients with many comorbidities and older age outside clinical trials. The emergence of such AEs and their management in clinical practice are critical for the translation of the survival advantages showed in the trials. Thus, RW reports are of extreme importance for the successful and safe use of this drug.

An important prospective, observational study was recently presented, and the reports of several RW retrospective studies have been published and are summarized in **Table 3**. A large RW prospective, observational study (ClinicalTrials.gov Identifier NCT03091127) was conducted in Europe and Israel and enrolled 701 RRMM patients treated with carfilzomib-based therapy (55% with KRd, 39% with Kd, and 6% with carfilzomib-based triplets containing dexamethasone and cyclophosphamide, pomalidomide, or daratumumab). High rates of response were reported in both the KRd (ORR: 83%, rate of at least a complete response [≥CR]: 31%) and Kd (ORR: 68%, ≥CR: 13%) arms, with no substantial differences compared to the responses observed in clinical trials. Regarding the toxicity profile, 24% of patients treated with KRd and 20% of those treated with Kd experienced G≥3 AEs, with 7% and 5% of vascular AEs, respectively. These rates were particularly low compared to those observed in the registration trials, considering that around 50% and 60% of patients respectively treated with KRd and Kd had a prior history of cardiovascular conditions. Serious AE (SAEs) occurred in around 33% of patients in both groups, and 3% and 8% of them were fatal in the KRd and Kd groups, respectively (56). The occurrence of SAEs was lower than that observed in the ASPIRE (65%) and ENDEAVOR (60%) trials and was likely biased due to a lower frequency of SAEs in the observational trial than in the interventional trials, despite the similar rates of fatal events (ASPIRE not available; ENDEAVOR 7%).

**Table 3:** Major clinical trials and real-world studies based on carfilzomib for the treatment of RRMM.

Study	Age*	Median lines (range)/prior exposure	ResponseORR/≥VGPR/≥CR	PFS/OS/DOT/TTNT	Toxicity, G≥3
**MAJOR CLINICAL TRIALS**					
**ASPIRE** (9, 55) KRd: N=396	64	2 (1-3)/ Bort-exposed: 65% Len-exposed: 20%	- 87%/70%/31%	mPFS: 26 mo mOS: 48.3 mo mDOT: 72 weeks (~16.5 mo)	- Neutropenia 30% - Thrombocytopenia 20% - Pneumonia 16% - Hypertension 6% - Heart failure 4% - Ischemic heart disease 4%
**ENDEAVOR** (53, 54) Kd: N=464	65	2 (1-2)/ Bort-exposed: 54% Len-exposed: 38%	- 77%/54%/13%	mPFS: 18 mo mOS: 47 mo mDOT: 40 weeks (~9.2 mo)	- Neutropenia 3% - Thrombocytopenia 12% - Pneumonia 7% - Hypertension 15% - Heart failure 6% - Ischemic heart disease 3%
**MAJOR REAL-WORLD STUDIES**					
**20150262 (NCT03091127), Terpos et al., 2020** (56) KRd: N=382 Kd: N=273	KRd: 65 Kd: 68	KRd: 1 (NA) Kd: 3 (NA)/ Bort-exposed: 97% Len-exposed: KRd 34%, Kd 68% ASCT: KRd 63%, Kd 44%	KRd: - 83%/66%/31% Kd: - 68%/42%/13%	mPFS: NA mOS: NA mDOT: - KRd 14.6 mo - Kd 7.5 mo	KRd: - Hematologic AEs 20% - Infection 14% - Vascular AEs 7% Kd: - Hematologic AEs 14% - Infection 16% - Vascular AEs 5%
**Rete Ematologica Pugliese, Mele et al., 2021** (57) KRd: N=130	66	1 (NA)/ Bort-exposed: 97% Len-exposed: 36%	- 79%/54%/37%	mPFS: 24 mo mOS: NA	- Hematologic AEs 9% - Infection 2% (any G 14%) - Cardiac AEs 3% (any G 11%) - SPM 3%
**Rocchi et al., 2021** (58) KRd: N=197	63	2 (NA)/ Bort-exposed: 96% Len-exposed: 45% (ref: 22%)	- 88%/50%/21%	mPFS: 19.8 mo 1-y OS rate: 80% mDOT: NA	- Neutropenia 21% - Infection 11% - Hypertension 6% - Heart failure 1%
**Davies et al., 2021** (52) KRd: N=218	64	NA (53% ≥3 lines)/ PI+IMiD-exposed: 67% (ref: 21%)	–	mDOT: 7 mo mTTNT: 8.7 mo	NA

*Age: median age.

RRMM, relapsed/refractory multiple myeloma; K, carfilzomib; R, Len, lenalidomide; d, dexamethasone; N, number; Bort, bortezomib; NA, not available; ref, refractory; PI, proteasome inhibitor; IMiD, immunomodulatory drug; ORR, overall response rate; ≥VGPR, at least a very good partial response; ≥CR, at least a complete response; PFS, progression-free survival; OS, overall survival; DOT, duration of treatment; TTNT, time to next treatment; mPFS, median PFS; mOS, median OS; mDOT, median DOT; mTTNT, median TTNT; mo, months; y, years; G, grade; AEs, adverse events; SPM, second primary malignancy.

The median DOT was 16.5 months in the KRd group (similar to that of 14.5 months in the ASPIRE trial) and 7.5 months in the Kd group (slightly shorter than that of 9.2 months in the ENDURANCE trial), even if the observational study included a population with a higher median number of prior lines (2 vs. 3 in the ASPIRE and ENDEAVOR trials) (53, 54, 56).

RW retrospective studies reported similar data. Two Italian RW experience studies with similar populations of respectively 130 and 197 consecutive RRMM patients treated with KRd reported high efficacy rates (ORR: 79% and 83%, ≥CR: 37% and 21%) and a median PFS of 20 and 24 months. The most common G≥3 toxicities were hematologic (9%), cardiac (1% and 3%), vascular (6%), and infections (2% and 11%) (57, 58). The PFS was shorter than that observed in the ASPIRE trial. This could be related to the selection of patients enrolled in the clinical trial, as compared with the selection performed in the RW study. Indeed, in the RW study, a higher proportion of patients had impaired renal function (creatinine clearance 30-50 ml/min in 16% of RW patients vs. 6% of clinical-trial patients) and high-risk cytogenetic abnormalities (27% of RW patients vs. 10% of clinical-trial patients).

An US Health Registry retrospective study by Davies et al. analyzed data from patients with ≥2 prior lines of treatment treated with several combinations of novel agents with the backbones Rd or Pd. In this study, a subgroup of patients was treated with the KRd triplet (n=218), showing an overall dismal prognosis, as compared with that in patients treated with daratumumab-Rd and other Rd-containing combinations (median DOT: 7 months) (52). The DOT was shorter than that in the ASPIRE trial (16.5 months). Davies et al. reported that 25% of patients treated with KRd had high-risk cytogenetics and that 42% of them had renal insufficiency, thus denoting a “difficult-to-treat population”.

The management of cardiovascular toxicity is crucial in RW practice. The correct identification of patients with higher risk of CVAEs is a key step to reduce the rates of cardiovascular toxicity. A SEER-Medicare data set analysis reported that a higher risk of cardiovascular toxicity was associated with patient-related risk factors such as age ≥75 years, history of cardiovascular disease, and obesity (59). Secondly, an accurate clinical follow-up is needed to promptly reduce or discontinue drug treatment after the occurrence of a CVAE.

Generally, in the majority of the aforementioned RW studies, CVAEs were less frequent than in clinical trials. A meta-analysis of 24 trials using carfilzomib showed an incidence of hypertension, heart failure, and ischemic heart disease of 12%, 4%, and ~2%, respectively (G≥3: 4%, 2.5%, and 1%, respectively) (60). In some of the cited RW experience studies (see **Table 3**), a lower rate of CVAEs than in clinical trials could be due to several factors. First, safety reports from clinical trials increased the attention of physicians regarding the cardiovascular risk associated with carfilzomib. This generated a bias of selection, with a lower rate of cardiovascular risk factors detected in RW patients eligible for carfilzomib-based therapy, as compared with clinical-trial patients. Surprisingly, data from the largest observational study reported by Terpos et al. did not show an increase in the rate of CVAEs, not even in a population of patients with a previous history of cardiovascular disease (56). Second, as reported by Rocchi at al., an accurate clinical follow-up monitoring of arterial blood pressure and cardiac function can reduce the incidence of high-grade CVAEs (e.g., following the recommendations by the European Myeloma Network and the Italian Society of Arterial Hypertension) (58, 61).

### 3.2 Ixazomib

Ixazomib was the first oral PI to be approved in combination with lenalidomide and dexamethasone (IRd) for the treatment of patients with RRMM, based on the results of the phase III TOURMALINE-MM1 clinical trial. In this trial, the IRd triplet showed superiority over Rd plus placebo in RRMM patients who received 1-3 prior lines of therapy, with an improvement in terms of PFS (median PFS: 20 vs. 15 months, HR 0.74, *P*=0.01), even without a great improvement in terms of ORR (78% vs. 72% with Rd) (11). This superiority was confirmed across all subgroups analyzed, including patients with 3 prior lines of therapy and patients with high-risk cytogenetics (median PFS: 21.4 months vs. 9.7 months).

After the introduction of IRd, several RW retrospective studies were conducted in clinical practice to verify the benefits of ixazomib (62–64) (see **Table 4**). Response rates were comparable or even higher in RW studies than in clinical trials (73%-88%), as well as PFS (24-27 months), except for a single RW report that showed a clearly inferior median PFS outcome (11 months), likely due to a more pretreated population (40% of patients received 3 prior lines of therapy) with high-risk features (38% of patients with high-risk cytogenetics vs. 19% in the TOURMALINE-MM1 trial) (64). In the RW setting, prior exposure to lenalidomide was associated with a shorter PFS, as compared with no exposure to lenalidomide (62, 63).

**Table 4:** Major clinical trials and real-world studies based on ixazomib.

Setting	Study	Age*	Median lines (range)/prior exposure	Response(ORR/≥VGPR/≥CR)	PFS/OS/DOT	Toxicity, G≥3
**MAJOR CLINICAL TRIALS**						
**RRMM patients**	**TOURMALINE-MM1** (11) IRd: N=360 (vs. placebo-Rd)	66	(1-3)/ Bort-exposed: 69% Len-exposed: 12%	- 78%/48%/14%	mPFS: 20.6 mo OS: NR (FU: 23 mo)	- Neutropenia 18% - Anemia 9% - Thrombocytopenia 12% - Rash 5% - Neuropathy 2% - Diarrhea 6% (any G 51%) - Nausea 2% (any G 31%) - Discontinuation 17%
**First line**	**TOURMALINE-MM2** (65) IRd: N=351 (vs. placebo-Rd: N=354)	73-74	–	IRd: - 82%/63%/26% Placebo-Rd: - 80%/48%/14%	IRd vs. placebo-Rd: -mPFS: 35 vs. 21 mo - OS: NR vs. 52 mo	- AEs 88% vs. 81%
**Maintenance,** **TE patients**	TOURMALINE-MM3** (66) I: N=395 (vs. placebo: N=261)	58	PI-exposed: 59% IMiD-exposed: 11% PI+IMiD-exposed: 30%	–	mPFS: 23 mo mOS: NA	- Neutropenia 5% - Anemia 4% - Thrombocytopenia 5% - Rash 2% - Pneumonia 7% - Diarrhea 3%
**Maintenance,** **NTE patients**	TOURMALINE-MM4** (67) I: N=425 (vs. placebo: N=281)	73	PI-exposed: 83% IMiD-exposed: 32% PI+IMiD-exposed: 15%	–	mPFS: 17.4 mo	- Neutropenia 2% - Thrombocytopenia 2% - Infection 6%
**MAJOR REAL-WORLD STUDIES**						
**RRMM patients**	**Terpos et al., 2020** (62) IRd: N=155	68	1 (1-7)/ Bort-exposed: 91% Carf-exposed: 91% Len-exposed: 17%	- 74%/35%/16%	mPFS: 27 mo mOS: NA	- Neuropathy 3% (any G 35%) - Pneumonia 8% - Discontinuation 9%
**RRMM patients**	**Cohen et al., 2020** (63) Ixa-based regimens: N=78 (IRd: 82%)	68	1 (1-6)/ Bort-exposed: 87% Carf-exposed: 5% Len-exposed: 26%	- 88%/49%/9%	mPFS: 24 mo mOS: NR	- Neutropenia 6% - Thrombocytopenia 5% - Anemia 6% - Diarrhea 3% - Neuropathy 3% (any G 17%) - Rash 4% - Pneumonia 3% - Discontinuation 11%
**RRMM patients**	**Hungarian Ixazomib Named Patient Program, Varga et al., 2019** (64) IRd: N=77	68	2 (1–3)/ Bort-exposed: 99%	- 80%/22%/12%	mPFS: 11 mo mOS: NA	- Neutropenia 8% - Anemia 5% - Thrombocytopenia 7% - Diarrhea 1% - Infection 5%
**First line**	J. Li et al., 2020*** (68) IRd: N=38 Other Ixa-based regimens: N=47	67	–	- 92%/58%/26%	mPFS: NR 1-y PFS rate: 85.5% mOS: NR 2-y OS rate: 67%	- Neutropenia 29% - Anemia 31% - Thrombocytopenia 18% - Rash 42% - Pneumonia 13% - Diarrhea 10%
**Maintenance,** ** NTE patients**	**Shen et al., 2021** (69) I: N=37****	63	Bort-exposed: 100%	–	mPFS: 16 mo mOS: NA	- Neutropenia 1% - Diarrhea 6%

*Age: median age. **The data reported refer to the ixazomib maintenance arm. ***The data reported refer to the IRd arm, in order to perform the comparison. ****The data reported refer to NDMM NTE patients, excluding RRMM patients.

RRMM, relapsed/refractory multiple myeloma; TE, transplant-eligible; NTE, non-transplant-eligible; I, Ixa, ixazomib; R, Len, lenalidomide; d, dexamethasone; N, number; Bort, bortezomib; PI, proteasome inhibitor; IMiD, immunomodulatory drug; ORR, overall response rate; ≥VGPR, at least a very good partial response; ≥CR, at least a complete response; PFS, progression-free survival; OS, overall survival; DOT, duration of treatment; mPFS, median PFS; mOS, median OS; mo, months; y, years; NR, not reached; FU, follow-up; NA, not available; G, grade; AEs, adverse events; NDMM, newly diagnosed multiple myeloma.

Ixazomib oral administration is convenient and appealing in light of a possible “all-oral” therapeutic approach. Moreover, ixazomib toxicity was acceptable in the TOURMALINE-MM1 trial, although AEs, which usually resolved within 3 months of therapy and included few G>3 events, were more frequent in the IRd group than in the Rd plus placebo group. The safety profile of ixazomib in the RW seemed to be comparable to that in the TOURMALINE-MM1 trial (**Table 4**).

Overall, the findings from the TOURMALINE-MM1 were confirmed in the RW in terms of efficacy and safety. In a scenario where mAbs and other triplets containing a PI plus Rd (VRd, KRd) seem to be more effective in terms of response rates and prolonged PFS (**Tables 3**, **4**), the residual role of ixazomib remains an important, unanswered question. A RW report compared the efficacy and tolerability profile of IRd to other Rd-based combinations (VRd and KRd). Interestingly, in the RW, IRd showed a better toxicity profile and a similar efficacy and survival outcome, as compared with VRd and KRd, especially in intermediate-fit and frail patients (70). Costs are another important issue. Data from the TOURMALINE-MM1 trial showed that IRd did not increase the burden of healthcare resource utilization compared to Rd, with no increase in the rate of hospitalization and relative costs (71).

Unfortunately, this survival advantage of the ixazomib-based triplet IRd vs. Rd in the RRMM setting did not translate into a benefit in first-line elderly NTE patients. Indeed, IRd failed to demonstrate a significant improvement in terms of PFS, as compared with placebo-Rd (HR 0.83, 95% CI 0.67-1.02, *P*=0.073), reducing its appeal as a first-line option (65). Apart from this, ixazomib was investigated as maintenance therapy, showing benefits, as compared with placebo, after ASCT in the TOURMALINE-MM3 trial (median PFS: 26 vs. 21 months) and after induction treatment in NTE patients in the TOURMALINE-MM4 trial (median PFS: 17.4 vs. 9.4 months) (67). Nonetheless, a recent meta-analysis indirectly compared lenalidomide vs. ixazomib maintenance treatment, showing a lack of benefit from ixazomib maintenance as compared with lenalidomide both in TE and NTE patients (72). Two ixazomib-based RW studies focused on front-line therapy (68) and maintenance therapy (69) in NTE patients are reported in **Table 4**.

In summary, ixazomib-based therapy may represent a good therapeutic option for the treatment of RRMM patients who are elderly or frail or who have cardiovascular comorbidities hindering the use of carfilzomib.

## 4 Monoclonal Antibodies

### 4.1 Daratumumab

Daratumumab is a mAb directed against CD38 that was approved as monotherapy and in combination with IMiDs (lenalidomide) and PIs (bortezomib) after outstanding results in several trials (**Table 5**). Daratumumab monotherapy was evaluated in a pooled analysis of two trials (SIRIUS and GEN501), which enrolled 148 patients. This analysis reported the efficacy of daratumumab monotherapy in heavily pretreated patients (most of whom were PI- and IMiD-exposed or -refractory, with a median number of 5 prior lines of therapy), with ORR of 30%, rate of at least a very good partial response (≥VGPR) of 14%, limited median PFS of 3.7 months, and median OS of 20 months (73). Overall, daratumumab as a single agent was quite well tolerated, with major safety concerns regarding hematologic AEs and infusion-related reactions (IRRs).

**Table 5:** Major clinical trials and real-world studies based on daratumumab for the treatment of RRMM.

Study	Age*	Median lines (range)/prior exposure	Response ORR/≥VGPR/≥CR	PFS/OS/DOT/DOR	Toxicity, G≥3
**MAJOR CLINICAL TRIALS**					
SIRIUS and GEN501 pooled analysis** (73) SIRIUS, Dara SA: N=106 GEN501, Dara SA: N=42	64	5 (4-7)/ Len-exposed: 98% Len-ref: 84% PI+IMiD-ref: 87%	- 30%/14%/5%	mPFS: 3.7 mo mOS: 20 mo mDOR: 8 mo	- Neutropenia 10% - Thrombocytopenia 14% - Anemia 18% - IRR (any G) 48%
**CASTOR** (74–76) Dara-Vd: N=498	64	2 (1–9)/ Len-exposed: 35% Len-ref: 17% Bort-exposed: 64%	- 85%/63%/30%	mPFS: 16.7 mo	- Neutropenia 14% - Thrombocytopenia 46% - Anemia 16% - Pneumonia 10% - PN 5% - SPM 6%
**POLLUX** (8, 77, 78) Dara-Rd: N=569	65	1 (1-11)/ Len-exposed: 17% Bort-exposed: 84% Bort-ref: 20%	- 93%/80%/56%	mPFS: 44.5 mo OS: 42 mo (56%)	- Neutropenia 55% - Thrombocytopenia 15% - Febrile neutropenia 6% - Pneumonia 15% - SPM 8%
**MAJOR REAL-WORLD STUDIES**					
**Canadian Myeloma Research Group database (CMRG-DB), LeBlanc et al., 2021** (79) N=710: - Dara SA: N=100; - Dara-Vd: N=143; - Dara-Rd: N=263	66-70	3 (2-11)	Dara SA: - 45%/21%/NA Dara-Vd: - 57%/28%/NA Dara-Rd: - 84%/58%/NA	Dara-SA: - mPFS 3.7 mo - mOS: 20 mo Dara-Vd: - mPFS 7.8 mo - mOS: 27 mo Dara-Rd: - mPFS: 26.6 mo - mOS: 32.9 mo	NA
**Lovas et al., 2019** (80) Dara SA: N=48 Dara-Vd: N=19 Dara-Rd: N=29	62	3 (1-12)/ Len-exposed: 77% Bort-exposed: 97%	Dara SA: - 46%/21%/NA Dara-Vd: - 79%/26%/NA Dara-Rd: - 81%/31%/NA	mPFS: - Overall 17 mo - Dara-Vd 6.6 mo - Dara-Rd NR (18 mo of FU)	- Neutropenia 2% -Thrombocytopenia 3% - Infection 15% (G5 10%) - IRR (any G) 16%
**GIMEMA Lazio Group, Vozella et al., 2021** (81) Dara SA: N=62	62	3 (2-8)/ Len-exposed: 90% Bort-exposed: 88%	46%/17%/5%	mPFS: 2.7 mo mOS: 22 mo	- Neutropenia 5% - Anemia 15% - Thrombocytopenia 12% - IRR 6% (any G 15%)
**Markovic et al., 2021** (82) Dara SA: N=44	65	4 (2-9)/ PI+IMiD-ref: 75%	37%/27%/NA	mPFS: 7.2 mo mOS: 7.8 mo	- Anemia 22% - Thrombocytopenia 9% - IRR 13% (any G 27%)
**Jullien et al., 2019** (83) Dara SA: N=41	68	4 (2-9)/ PI+IMiD-ref: 58%	24%/5%/0%	mPFS: 1.9 mo mOS: 6.5 mo	- Anemia 12% - Thrombocytopenia 12% - Infection 10% - IRR 3% (any G 29%)
**Polish Myeloma Group, Salomon-Perzyński et al., 2019** (84) Dara SA: N=30	63	4 (2-10)/ PI+IMiD-ref: 50%	43%/28%/21%	mPFS: 9.5 mo mOS: 13.8 mo	- Neutropenia 13% - Anemia 16% - Pneumonia 10% - IRR 6% (any G 20%)
**Optum’s deidentified electronic health record (EHR) database, Davies et al., 2021** (52) Dara-Rd: N=99 Dara-Pd: N=149	Dara-Rd: 70 Dara-Pd: 68	NA/ Dara-Rd: 42% ≥4 lines Dara-Pd: 72% ≥4 lines PI+IMiD-exposed: 67% and 86%, respectively (ref: 31% and 68%, respectively)	–	mDOR: - Dara-Rd: NA - Dara-Pd: 6 mo mTTNT: - Dara-Rd: NA - Dara-Pd: NA	NA

*Age: median age. **Different treatment schedules were included in the phase I/II GEN501 trial. For the sake of clarity, all information regarding the different groups is reported together.

RRMM, relapsed/refractory multiple myeloma; Dara, daratumumab; SA, as a single agent; V, Bort, bortezomib; d, dexamethasone; N, number; R, Len, lenalidomide; GIMEMA, Italian Adult Haematological Diseases Group; P, pomalidomide; PI, proteasome inhibitor; IMiD, immunomodulatory drug; ref, refractory; ORR, overall response rate; ≥VGPR, at least a very good partial response; ≥CR, at least a complete response; NA, not available; PFS, progression-free survival; OS, overall survival; DOT, duration of treatment; DOR, duration of response; mDOR, median DOR; mPFS, median PFS; mOS, median OS; mDOT, median DOT; mo, months, FU, follow-up; NR, not reached; G, grade; IRR, infusion-related reaction; PNP, peripheral neuropathy; SPM, second primary malignancy.

Compared with the findings from clinical trials, several small RW retrospective studies exploring daratumumab monotherapy (**Table 5**) included patients with a similar or often lower number of prior lines of therapy and showed consistent or even superior rates of efficacy. Overall, a substantial difference between trial and RW safety reports was observed in terms of the incidence of IRRs, which represents an important issue in the use of daratumumab in clinical practice. IRRs of any grade were variably reported in 15%-30% of cases in the RW setting, as compared with 48% in the SIRIUS and GEN501 trials. Nonetheless, in the RW, G≥3 IRRs varied from 3% to 13%, and it was not clear if they were more frequent in clinical practice. Differently from daratumumab monotherapy, in the RW setting, only few data are available on the combination of this drug with lenalidomide, pomalidomide, bortezomib, and dexamethasone (Dara-Rd, Dara-Pd, and Dara-Vd, respectively). As already mentioned, Davies et al. reported data (with few survival data, due to the short follow-up) on 99 patients treated with Dara-Rd and 149 with Dara-Pd who were heavily pretreated (median of 4 prior lines of therapy) (52).

A study based on the Canadian Myeloma Research Group database (CMRG-DB) evaluated efficacy and toxicity in 710 patients treated with daratumumab as a single agent or in combination with Rd or Vd. Responses were higher with Dara-Rd than with Dara-Vd and daratumumab monotherapy (ORR: 85% vs. 58% vs. 45%; ≥VGPR: 59% vs. 29% vs. 21%), thus confirming the synergistic effect of daratumumab plus lenalidomide also in clinical practice. Survival data were also more positive with Dara-Rd (median PFS of 26 months and median OS of 32 months), as compared with the other two treatment regimens (79).

An important issue in the use of daratumumab is the speed of infusion. Indeed, there is RW evidence that a rapid daratumumab infusion protocol did not increase the rate of AEs, as compared with the standard infusion protocol (85, 86).

### 4.2 Elotuzumab

Elotuzumab (Elo) is a mAb targeting the glycoprotein receptor SLAMF7, triggering the activation of natural killer cells and myeloma cell death through antibody-dependent cellular cytotoxicity (ADCC). Elotuzumab failed to demonstrate significant anti-tumor activity as a single agent, but showed good results in combination with Rd (Elo-Rd) and Pd (Elo-Pd) (87). In the randomized, phase III ELOQUENT-2 trial, Elo-Rd vs. Rd showed a better ORR (79% vs. 66%) and a longer PFS (median PFS: 19 vs. 15 months). The number of previous lines of treatment did not affect therapy outcome. Similarly, no differences were observed between patients with standard-risk and high-risk cytogenetics (88). The main safety concerns were infections (G≥3: 35% vs. 27% with Rd, G≥3 pneumonia: 15% vs. 10% with Rd) and hematologic AEs (**Table 6**) (88).

**Table 6:** Major clinical trials and real-world studies based on elotuzumab for the treatment of RRMM.

Study	Age*	Median lines (range)/prior exposure	ResponseORR/≥VGPR/≥CR	PFS/OS	Toxicity, G≥3
**MAJOR CLINICAL TRIALS**					
ELOQUENT-2** (10, 88) Elo-Rd: N=321 (vs. Rd: N=325)	66	2 (1-3)/ Bort-exposed: 47% Len-exposed: 5%	- 79%/33%/4%	mPFS: 19.4 mo mOS: 48.3 mo	- Neutropenia 27% - Anemia 18% - Infections 35% - Pneumonia 15% - Fatigue 10% - Cardiac disorders 6%
ELOQUENT-3** (49) Elo-Pd: N=60 (vs. control group: N=57)	69	3 (2-8)/ Bort-exposed: 100% Len-exposed: 98% Len+Bort-ref: 68%	- 53%/20%/8%	mPFS: 10.3 mo mOS: NR	- Anemia 10% - Neutropenia 13% - Thrombocytopenia 8% - Infection 13% - Pneumonia 5% - Cardiac disorders 7%
**MAJOR REAL-WORLD STUDIES**					
**Gentile et al., 2021** (89) Elo-Rd: N=300	NA	1 (1-4)/ Bort-exposed: 94% Len-exposed: 26%	- 77%/29%/8%	mPFS: 17.6 mo mOS: NR 1-y OS rate: 66%	- Neutropenia 19% - Anemia 16% - Thrombocytopenia 10% - Infections 34% - Pneumonia 17% - Fatigue 21%
**University hospitals of Würzburg and Vienna, Hose et al., 2021** (90) Elo-Pd: N=22	62	5 (1-16)/ Len-exposed: 100% Len-ref: 59% Poma-exposed: 68%	- 50%/NA/NA	mPFS: 6.4 mo mOS: NR 1-y OS rate: 82%	- Neutropenia 13% - Thrombocytopenia 4% - Pneumonia 9%

*Age: median age. **Data reported refer to the elotuzumab arm.

RRMM, relapsed/refractory multiple myeloma; Elo, elotuzumab; R, Len, lenalidomide; d, dexamethasone; P, Poma, pomalidomide; N, number; Bort, bortezomib; ref, refractory; ORR, overall response rate; ≥VGPR, at least a very good partial response; ≥CR, at least a complete response; NA, not available; PFS, progression-free survival; OS, overall survival; mPFS, median PFS; mOS, median OS; mo, months; y, years; NR, not reached.

A large Italian retrospective multicenter RW study investigated the use of Elo-Rd in 300 RRMM patients treated outside controlled clinical trials, with rates of ORR, PFS, and OS comparable to those observed in the ELOQUENT-2 trial. Of note, RW patients had received 1 median previous line of therapy, as compared with 2 previous lines in the clinical trial. By contrast, 25% of RW patients was already exposed to lenalidomide, as compared with 5% in the ELOQUENT-2 trial. Indeed, treatment response was suboptimal among patients previously exposed to lenalidomide, suggesting that lenalidomide exposure is of key importance; the ELOQUENT-2 clinical trial provided information about this subset of patients. Safety data were superimposable between RW and trial patients (**Table 6**) (89).

A great limitation to the use of Elo-Rd in clinical practice may be the continuous use of lenalidomide as part of the first-line regimen (either as maintenance treatment after ASCT or in combination with daratumumab and dexamethasone in NTE patients). In fact, a considerable proportion of patients develop lenalidomide-resistant disease.

Recently, the multicenter, randomized, phase II ELOQUENT-3 trial assessed the efficacy of Elo-Pd vs. Pd in RRMM patients who had previously received treatment with lenalidomide and a PI. Elo-Pd vs. Pd was associated with higher ORR and longer PFS, even across several subgroups of patients with high-risk ISS disease and/or abnormal cytogenetics and/or more than 4 prior lines of therapy.

The addition of elotuzumab did not increase the rate of AEs, as compared with Pd. Infections were less common in the Elo-Pd group than in the control group, as well as neutropenia and anemia (49). These good and promising safety and efficacy results were confirmed by a small RW retrospective report on 22 patients. Interestingly, the median PFS in pomalidomide-exposed patients was identical to that observed in pomalidomide-naïve patients, suggesting that the previous administration of pomalidomide-based regimens may not be a limitation (90).

In the first-line setting, similarly to ixazomib, elotuzumab in combination with Rd failed to demonstrate an improved outcome, as compared with the standard-of-care Rd. Even though results have not yet been published, a report from the sponsor of the phase III ELOQUENT-1 trial (NCT01335399) explained that this trial did not meet the primary endpoint of improved PFS in elderly untreated patients (91). This limits the use of the Elo-Rd triplet in the first-line treatment, particularly in light of the results of the MAIA trial comparing Dara-Rd vs. Rd.

## 5 Novel Agents: Small Molecules and Immunotherapeutic Approaches

### 5.1 Venetoclax

Venetoclax is an oral inhibitor of the anti-apoptotic protein Bcl-2, represents a standard of care in chronic lymphocytic leukemia and acute myeloid leukemia, and showed preliminary encouraging results in MM (92). The phase III BELLINI trial compared venetoclax plus Vd vs. placebo plus Vd in RRMM patients. Even though the median PFS and the ORR were higher in the venetoclax group than in the placebo group, the incidences of treatment-emergent AEs and SAEs were similar in the two groups, and there was an imbalance in the number of deaths favoring the placebo group. This is probably related to the immunosuppressive effect of venetoclax. However, in a subgroup analysis, the authors showed that patients with translocation t(11;14) or high Bcl-2 gene expression showed improved responses and PFS without the increased mortality rate associated with venetoclax administration (74 patients in the venetoclax group vs. 40 patients in the placebo group: PFS, NR vs. 9.9 months; ORR, 85% vs. 70%; **Table 6**) (93).

RW data on the use of venetoclax are scarce and based on the analysis of small groups of patients. Efficacy was confirmed in a RW series of 10 patients harboring t(11;14) who were treated with venetoclax combined with dexamethasone, bortezomib, carfilzomib, or daratumumab. Despite the small number of patients, this study confirmed the potential toxicity of venetoclax, which was mainly related to hematologic AEs and enhanced infection susceptibility and required transfusion support (94).

Another RW retrospective study was conducted on 11 patients without t(11;14). The ORR was low, and the median PFS was of 2 months (95). This report confirmed the toxicity profile of venetoclax, which will hopefully be explored in the t(11;14) setting, as in the ongoing phase III M13-494 (CANOVA) trial (NCT03539744).

### 5.2 Selinexor

Selinexor is an oral selective inhibitor of exportin 1 (XPO1; a nuclear export protein overexpressed in MM cells) leading to the activation of tumor suppressor proteins. Currently, selinexor has been approved by the Food and Drug Administration (FDA) and is awaiting the approval of the European Medicines Agency (EMA), based on the phase IIb, multicenter, open-label STORM clinical trial. In this study, 122 RRMM patients were treated with selinexor plus dexamethasone (sel-dex). The majority of patients were penta-exposed (exposed to 2 PIs, 2 IMiDs, and 1 anti-CD38 mAb) or triple-class refractory (refractory to 1 PI, 1 IMiD, and 1 anti-CD38 mAb). The ORR was 26%, with a median PFS of 3.7 months and a median OS of 8.6 months. Although these results may appear disappointing, these patients had few other therapeutic options (96). Moreover, these findings were corroborated by the comparison with data on an observational cohort of penta-exposed RRMM patients in the Flatiron Health Analytic Database (FHAD) who were treated in the RW setting with other therapies. Baseline characteristics and demographics were comparable, although patients in the STORM cohort were more heavily pretreated. Despite the methodological limitations of this analysis, the OS was significantly better in triple-class-refractory MM patients treated with sel-dex than in those treated with the other available therapies (97).

In the STORM trial, G≥3 hematologic toxicities were frequent, especially thrombocytopenia, anemia, fatigue, and nausea. Overall, 18% of patients discontinued treatment due to an AE. Of note, hyponatremia occurred in 37% of patients (G≥3: 21%) (96). In a RW retrospective analysis of a single-center experience, hyponatremia was reported in 13 out of 17 patients within 5 weeks of therapy, 8 out of 13 patients required hospitalization, 3 patients had G3 hyponatremia with severe symptoms, and 1 patient was admitted to intensive care. The incidence was higher compared with clinical trial data, probably due to a higher presence of comorbidities (advanced chronic kidney disease and heart failure). For this reason, the authors suggest more frequent urine analyses and early referral to nephrology care for the evaluation and treatment of hyponatremia and the prevention of potentially serious symptoms (98).

### 5.3 Belantamab Mafodotin

Belantamab mafodotin (GSK2857916) is an antibody–drug conjugate (ADC) targeting B-cell maturation antigen (BCMA). In the phase I DREAMM-1 and phase II DREAMM-2 trials, it showed single-agent activity in heavily pretreated RRMM patients. In the DREAMM-2, the ORR was 30%, with a median PFS of 2.8 months, while the median OS was NR at 6 months of follow-up (99). The main AEs were cytopenias such as thrombocytopenia and corneal toxicity, especially keratopathy, which was already reported with the use of an ADC conjugated with monomethyl auristatin F (MMAF) (100).

Currently, there are no large RW studies on belantamab mafodotin, except one which was conducted on 32 RRMM patients treated in 6 medical Israeli centers, confirming the results from the DREAMM-2 study in terms of rate of response and main AEs (101) (**Table 7**).

**Table 7:** Major clinical trials and real-world studies based on other novel agents (monoclonal antibodies and small molecules) for the treatment of RRMM.

Study	Age*	Median lines (range)/prior exposure	Response(ORR/≥VGPR/≥CR)	PFS/OS	Toxicity, G≥3
**MAJOR CLINICAL TRIALS**					
*Venetoclax-based:* **BELLINI** (93) Ven-Vd: N=194	66	NA (range 1-3)/ PI-exposed: 61% IMiD-exposed: 40%	- 82%/59%/26%	mPFS: 22.4 mo mOS: NR	- Neutropenia 18% - Thrombocytopenia 14%- Pneumonia 14%- Treatment-related death 1.5%
*Belamaf-based:* **DREAMM-1** (102) Belamaf 3.4 mg/kg: N=35	60	NA (57% received ≥5 lines)/ PI-exposed: 100% IMiD-exposed: 100%	- 60%/51%/8%	mPFS: 7.9 mo	- Corneal event 3%- Thrombocytopenia 48%- Anemia 14%- Neutropenia 3%- Pneumonia 6%
*Belamaf-based:* **DREAMM-2** (99) Belamaf 2.5 mg/kg: N=97	65	7 (3-21)/ Bort-exposed: 97% Len-exposed: 100%	- 30%/20%/NA	mPFS: 2.9 mo mOS: NR (6 mo of FU)	- Keratopathy 27%- Thrombocytopenia 19%- Anemia 20%- Pneumonia 4%- Neutropenia 9%
**MAJOR REAL-WORLD STUDIES**					
*Venetoclax-based:* **Basali et al., 2020** (94) Ven-d: N=1 Ven-Vd: N=6 Ven-Kd: N=2 Ven-Dara-Vd: N=1	NA	6 (2-19)	- 78%/NA/NA	6-mo PFS rate: 28%6-mo OS rate: 77%	- Blood transfusion due to hematologic toxicities 40% - Death due to septic shock 10%
*Venetoclax-based:* **Jelinek et al., 2020** (95)Ven-Vd: N=11	69	7 (4-10)/ Len- and Bort-exposed: 100%	- 27%/NA/NA	mPFS: 2 mo mOS: 12 mo	- Infection 27%- Thrombocytopenia 46%- Nausea 27%
*Belamaf-based:* **GSK^®^ Expanded Access Program, Shragai et al., 2020** (101) Belamaf 3.4 mg/kg: N=17 Belamaf 2.5 mg/kg: N=15	69.6	6 (3-11)/ Bort-exposed: 94% Len-exposed: 91% Carf-exposed: 74% Dara-exposed: 97%	- 43%/32%/3%	mPFS: 2.6 mo 6-mo OS rate: 68%	- Ocular toxicities 26%- Thrombocytopenia 13%- Neutropenia 17%- Infection 13%

*Age: median age.

RRMM, relapsed/refractory multiple myeloma; Ven, venetoclax; V, Bort, bortezomib; d, dexamethasone; belamaf, belantamab mafodotin; K, Carf, carfilzomib; Dara, daratumumab; GSK^®^, GlaxoSmithKline^®^; NA, not available; PI, proteasome inhibitor; IMiD, immunomodulatory drug; Len, lenalidomide; PFS, progression-free survival; OS, overall survival; mPFS, median PFS; mOS, median OS; mo, months; y, years; NR, not reached; FU, follow-up; G, grade.

## 6 Discussion

In conclusion, the availability of multiple options for the treatment of NDMM and RRMM patients allows to improve the treatment decision process, even though some considerations about efficacy and safety data from randomized clinical trials should be taken into account. A RW study of RRMM patients highlighted some reasons for ineligibility in RCTs: age, prior lines of therapy with disease refractory to previous treatments, renal failure, and comorbidities, with particular reference to cardiovascular concomitant disease (103). These concerns confirm the discrepancies that can be observed between RW and RCT regimens in terms of safety, quality of life, and efficacy. Regimens that are more tolerated and do not impact negatively on patients’ quality of life may be administered for longer periods, thus improving their efficacy. Moreover, differences in terms of national policies and local availabilities of drugs may impact on the gap among RCTs and RW.

Prospective multinational studies exploring standards of care in the RW setting are ongoing (4, 104), and new studies are still needed to detect all possible discrepancies between clinical trials and RW practice, together with the validation of new tools or composite metrics incorporating these additional considerations. This process can foster comparisons across different regimens for the treatment of RW patients, thus leading to patient-focused decision making and treatment-tailored approaches. This is particularly true for patients with triple-class refractoriness, who represent a high unmet medical need especially in the RW setting, thus stressing the necessity of the approval and routine use of new potent drugs. For instance, in the phase Ib/II CARTITUDE-1 study, ciltacabtagene autoleucel was associated with significantly improved responses and outcomes, as compared with the RW results from the LocoMMotion prospective study (105).

## Author Contributions

Substantial contributions to the conception or design: all authors. Interpretation of data: all authors. First draft: all authors. Supervision: SO. Critical revision for important intellectual content: all authors. Agreement to be accountable for all aspects of the work in ensuring that questions related to the accuracy or integrity of any part of the work are appropriately investigated and resolved: all authors. All authors contributed to the article and approved the submitted version.

## Conflict of Interest

Author SO has received honoraria from Amgen, Celgene/Bristol Myers Squibb, and Janssen; has served on the advisory boards for Adaptive Biotechnologies, Janssen, Amgen, and Takeda.

The remaining authors declare that the research was conducted in the absence of any commercial or financial relationships that could be construed as a potential conflict of interest.

## Publisher’s Note

All claims expressed in this article are solely those of the authors and do not necessarily represent those of their affiliated organizations, or those of the publisher, the editors and the reviewers. Any product that may be evaluated in this article, or claim that may be made by its manufacturer, is not guaranteed or endorsed by the publisher.
